# An *In Vitro* Platform to Study Reversible Endothelial-to-Mesenchymal Transition

**DOI:** 10.3389/fphar.2022.912660

**Published:** 2022-06-23

**Authors:** Muthu Kumar Krishnamoorthi, Rajarajan A. Thandavarayan, Keith A. Youker, Arvind Bhimaraj

**Affiliations:** Houston Methodist Research Institute, Houston Methodist Hospital, Houston, TX, United States

**Keywords:** endothelial cells, L-NAME, angiotensin II, endothelial-to-mesenchymal transition, reversible EndoMT, endothelial functionality, TGF-β1

## Abstract

Endothelial cells can acquire a mesenchymal phenotype in response to external stimuli through both mechanical and biological factors, using a process known as endothelial-to-mesenchymal (EndoMT) transition. EndoMT is characterized by the decrease in endothelial characteristics, increase in mesenchymal markers, and morphological changes. It has been recognized not only during development but also in different pathological conditions including organ/tissue fibrosis in adults. The ability to modulate the EndoMT process could have a therapeutic potential in many fibrotic diseases. An *in vitro* method is presented here to induce EndoMT with Nω-nitro-L-arginine methyl ester hydrochloride (L-NAME) and angiotensin II (Ang II) followed by a protocol to study the reversibility of EndoMT. Using this method, we furnish evidence that the combination of L-NAME and Ang II can stimulate EndoMT in Human umbilical vascular endothelial cells (HUVECs) and this process can be reversed as observed using endothelial functionality assays. This method may serve as a model to screen and identify potential pharmacological molecules to target and regulate the EndoMT process, with applications in drug discovery for human diseases.

## 1 Introduction

Endothelial-to-mesenchymal transition (EndoMT) is a dynamic process in which endothelial cells undergo complex molecular changes through which they lose their endothelial attributes and acquire a mesenchymal cell-like phenotype (Sánchez-Duffhues et al., 2018), a form of epithelial-to-mesenchymal (EMT) transition (Zeisberg et al., 2007). This allows the well-ordered endothelial cells to differentiate into spindle-shaped mesenchymal-like cells. Morphological alterations are accompanied with changes in protein expression. In general, loss of endothelial markers [e.g., Platelet endothelial cell adhesion molecule-1 (PECAM-1) or cluster of differentiation 31 (CD31)] and simultaneous acquisition of mesenchymal attributes (e.g., Vimentin) are observed.

EndoMT was originally illustrated in cardiac development (Markwald et al., 1975) and has since been indicated to be a source for other cell types (cardiac pericytes and vascular smooth muscle cells) of the vascular tree (Chen et al., 2016) and in facilitating angiogenesis (Welch-Reardon et al., 2015). More recent evidence suggests that EndoMT (Zeisberg et al., 2007; Zhang et al., 2016) and its reversal (Tombor et al., 2021) are also associated with cardiovascular disease processes including recovery. Manipulation of EndoMT-associated cellular pathways has been shown to mitigate pathological settings in cardiac fibrosis in animal models (Zeisberg et al., 2007).

Recently our group has reported evidence on the role of endothelial and mesenchymal cell transitions during heart failure (HF) and the subsequent “natural” recovery, in a non-ischemic mouse model of heart failure (Wang et al., 2020). In this model, the presence and removal of Nω-nitro-L-arginine methyl ester hydrochloride (L-NAME), salt and the use of an osmotic pump to supply angiotensin II (Ang II) result in HF and natural recovery, respectively. Ang II infusion is a widely used approach to induce HF in animal models (Tsukamoto et al., 2013; Cambier et al., 2018; Aghajanian et al., 2019; Wang et al., 2020) by increasing blood pressure and L-NAME, a nitric oxide synthase antagonist (Liu et al., 2019), and salt further contributes to HF. Both L-NAME and Ang II have been independently reported to induce EndoMT *in vitro* (Gao et al., 2020; Tombor et al., 2021).

**FIGURE 1:**
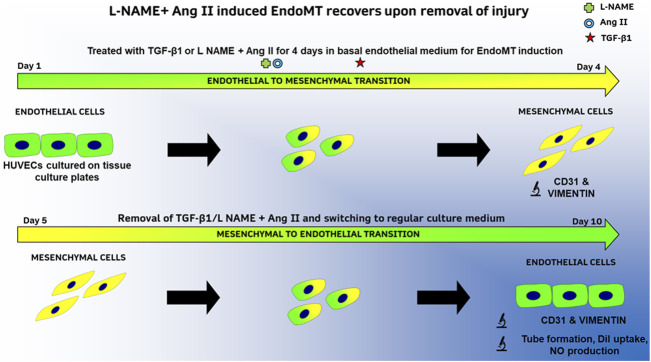
Summary of methods to study EndoMT and its reversal in HUVECs with TGF-β1 or L-NAME + Ang II.

In parallel to the established animal model described above, we describe here an *in vitro* method to induce and document EndoMT and its reversal with a combination of L-NAME and Ang II. TGF-β1 is used as a positive control (as summarized in Figure 1). EndoMT hallmark investigations like protein expression (up and downregulation of markers), morphological changes, and cytoskeletal rearrangements were performed to validate this methodology. We also report for the first time, functional recovery of endothelial cells after EndoMT *in vitro*. This method could serve as an *in vitro* platform to both understand EndoMT mechanisms and as a drug testing platform to identify and validate pharmaceutical molecules that can either inhibit EndoMT or affect recovery after EndoMT. This recovery is likely a form of mesenchymal-to-endothelial transition (MEndoT).

## 2 Methods

The materials and equipment used in this study are listed in Tables 1, 2, respectively.

**TABLE 1:** List of materials.

1	4-amino-5-methylamino-2′,7′-difluororescein diacetate—DAF-FM™ diacetate (Invitrogen, D-23844)
2	4′,6-diamidino-2-phenylindole—DAPI (IHCWORLD, IW-1404)
3	8 chambered slides (ThermoScientific, 154534)
4	15 ml conical centrifuge tubes (ThermoScientific, 339651)
5	96-well plates, nonsterile (BD Falcon, 353915)
6	alamarBlue™ Cell Viability Reagent (ThermoFisher, DAL1025)
7	Alexa Fluor 488 donkey anti-goat IgG (ThermoFisher, A-11055)
8	Angiotensin II—Ang II (Sigma Aldrich, A9525-10MG)
9	Anhydrous dimethyl sulfoxide—DMSO (Invitrogen, D12345)
10	Bovine serum albumin—BSA (Millipore, 126575-10GM)
11	Dil conjugated Acetylated Low-Density Lipoprotein—DiI AcLDL (Invitrogen, L3484)
12	Disposable sterile plastic pipettes
13	Dulbecco’s Phosphate Buffered Saline—DPBS (Gibco, 14190-144)
14	EGM^TM^-2 Endothelial Cell Growth Medium-2 BulletKit^TM^ (Lonza, CC-3162)
15	EBM^TM^-2 Basal Medium (Lonza, CC-3156)
16	EGM^TM^-2 SingleQuots^TM^ Supplements (Lonza, CC-4176)
17	Hanks Balanced Salt Solution with calcium and magnesium—HBSS (Gibco, 14025092)
18	Human umbilical vascular endothelial cells - HUVEC (Lonza, C2519A)
19	Hydrochloric acid—HCl (Sigma Aldrich, 339253-100ML)
20	Matrigel^®^, growth factor reduced, LDEV free (Corning, 356230)
21	Methanol (Sigma Aldrich, 34860-4L-R)
22	Nω-Nitro-L-arginine methyl ester hydrochloride—L-NAME (Sigma Aldrich, N5751-10G)
23	PECAM-1 goat polyclonal affinity purified antibody—CD31 (Santa Cruz, sc-1506)
24	Recombinant Human TGF-β1 (Peprotech, 100-21-2UG)
25	Rhodamine Phalloidin (Cytoskeleton Inc., PHDR1)
26	Syringe filter, 0.2 µm (Fisher, 09-719C)
27	Tissue culture plates
	• 6-well plates (Corning Falcon, 353046)
	• 48-well plates (Corning Falcon, 353078)
	• 24-well plates (Corning Costar, 3526)
28	Triton X-100 (RPI, T60085-1)
29	Transforming Growth Factor-β1—TGF-β1 (Peprotech, 100-21-2UG)
30	Tryphan blue solution (Gibco, 15250061)
31	Trypsin EDTA (Gibco, 25300054)
32	UltraCruz^®^ Blocking Reagent (Santa Cruz, sc-516214)
33	Vimentin mouse monoclonal antibody (Santa Cruz, sc-6260 TRITC conjugated)

**TABLE 2:** List of equipment.

1	Cell culture incubator (humidified, 5% CO_2_)
2	Biological hood with laminar flow and UV light
3	Pipette aid
4	Sterile micropipette
5	37^°^C water bath
6	Centrifuge
7	Cell counter (Countess II FL)
	• Cell counting slides (Invitrogen, C10283)
8	Inverted phase microscope with fluorescence and ×4 and ×20 objectives (EVOS M5000)
9	Microplate reader (TECAN Spark)

### 2.1 Experimental Procedure

#### 2.1.1 Reagent Preparation

*•* Endothelial cell growth medium preparation*:* Add all supplements and growth factors of the EGM^TM^-2 SingleQuots Kit to EBM^TM^-2 and store at 4^°^C for up to 1 month.
• Basal medium preparation: Add only the FBS (10 ml) and GA-1000 (0.5 ml) of EGM^TM^-2 SingleQuots^TM^ Supplements to EBM^TM^-2 Basal Medium and store at 4^°^C for up to 1 month.
• TGF-β1: Add 0.5 ml of HCl/BSA solution (4 mM HCl containing 1 mg/ml BSA) to TGF-β1 vial and vortex briefly (30 s). Add another 0.5 ml of HCL/BSA solution and centrifuge to collect the liquid. Prepare 50 µl working aliquots in sterile tubes and store at –80^°^C. Add 5 µl of TGF-β1 solution to 1 ml of EBM^TM^-2 media to get a final concentration of 10 ng/ml.
• L-NAME: Add 0.267 g of L-NAME powder to 1 ml of sterile water and filter through a sterile syringe filter to make a 1 M solution. Prepare 50 µl working aliquots in sterile tubes and store at –80^°^C. Add 1 µl of 1 M solution to 1 ml of EBM^TM^-2 media to get a final concentration of 1 mM.
• Ang II: Add 956 µl of sterile water to one vial of 10 mg Angiotensin to get a stock solution of 1 mM. Prepare 50 µl working aliquots in sterile tubes and store at –80^°^C. Add 1 µl of 1 mM Ang II solution to 1 ml of EBM^TM^-2 media to get a final concentration of 1 µM.
• DAF-FM™ diacetate: Add 20 μl of high-quality anhydrous DMSO to the 50 μg packaging to make a ∼5 mM stock solution (As per the manufacturer’s recommendation). Add 1.6 µl of 5 mM stock to 1 ml of media to obtain a final concentration of 8 µM.
• Rhodamine Phalloidin: Add 500 µl of 100% methanol to the lyophilized powder to prepare a 14 µM solution (×200 stock) (as per the manufacturer’s recommendation)
• Blocking buffer: Add 1 ml of UltraCruz^®^ Blocking Reagent and 0.05% of Triton X-100 to 10 ml of 1X DPBS.
• DiI AcLDL: Add 1 µl of stock in 200 µl media to prepare a 1:200 dilution working solution.
• alamarBlue™ Cell Viability Reagent: Prepare a working solution of 10% (v/v) solution with the corresponding cell culture media (EBM^TM^-2 or EGM^TM^-2).

#### 2.1.2 Passaging of Human Umbilical Vascular Endothelial Cells—(Time for Experiment—30 min) (2 Days Before Endothelial-to-Mesenchymal Transition Induction)

a) Maintain HUVEC cells (Passage 2–5) in standard culture conditions in complete EGM^TM^-2 media and avoid 100% confluency.
b) Wash HUVECs plated in 6-well plates with 1X DPBS and add 1 ml of trypsin EDTA to each well and incubate for 4 min at 37^°^C.
c) Detach cells using 4 ml of culture media and collect cell suspension in 15 ml tubes.
d) Centrifuge the cell suspension at 200–300 × g for 4 min at room temperature.
e) Carefully aspirate the supernatant and suspend the cell pellet in pre-warmed culture media.
f) Count viable cells using trypan blue solution in the automatic cell counter (or a standard hemocytometer).
g) Plate 2.0 × 10^5^ to 4.0 × 10^5^ HUVECs onto each well of a 6-well tissue culture plate, such that the cells will be ∼80% confluent in 24 h.

#### 2.1.3 Preparation of Human Umbilical Vascular Endothelial Cells for Endothelial-to-Mesenchymal Transition Induction–(Time for Experiment—15 min) (24 h Before Endothelial-to-Mesenchymal Transition Induction)

a) When cells are ∼80% confluent, replace complete EGM^TM^-2 with basal EBM^TM^-2 media.

#### 2.1.4 Induction of Endothelial-to-Mesenchymal Transition in Human Umbilical Vascular Endothelial Cells by Transforming Growth Factor-β1 or Nω-Nitro-L-Arginine Methyl Ester Hydrochloride + Angiotensin II—(Time for Experiment—4 Days)

a) Prepare HUVECs following steps 2 and 3.
b) For EndoMT induction with TGF-β1, add 3 ml of EBM^TM^-2 media containing 10 ng/ml of TGF-β1 to HUVECs.
c) For EndoMT induction with L-NAME + Ang II, add 3 ml of EBM^TM^-2 media containing 1 mM L-NAME and 1 µM Ang II to HUVECs.
d) Replace the media with fresh pre-warmed EBM^TM^-2 media containing either 10 ng/ml of TGF-β1 or 1 mM L-NAME and 1 µM Ang II after 48 h
e) Proceed to subsequent analyses to evaluate F-actin reorganization and change in endothelial and mesenchymal markers.

#### 2.1.5 Recovery of Endothelial-to-Mesenchymal Transition in Human Umbilical Vascular Endothelial Cells Induced by Transforming Growth Factor-β1 and/or Nω-Nitro-L-Arginine Methyl Ester Hydrochloride/Angiotensin II—(Time for Experiment—6 Days)

a) Prepare HUVECs following steps 2, 3, and 4.
b) After treatment with either 10 ng/ml of TGF-β1 or 1 mM L-NAME and 1 µM Ang II for 4 days to induce EndoMT, wash cells with 1X DPBS.
c) Replace the media with fresh pre-warmed complete EGM^TM^-2 media. Replenish media every 48 h for up to 6 days.
d) Proceed to subsequent analyses to evaluate change in endothelial and mesenchymal markers.

#### 2.1.6 Immunocytochemical Analysis

a) Plate 1.0 × 10^4^ to 4 × 10^4^ HUVEC cells onto each well of an 8-chambered slide. Prepare slides for EndoMT induction following step 3.
b) Proceed with step 4 of EndoMT induction with either 10 ng/ml of TGF-β1 or 1 mM L-NAME and 1 µM Ang II for 4 days.
c) F-Actin staining—(time for experiment—1 day)
  1) After 4 days of EndoMT induction by either 10 ng/ml of TGF-β1 or 1 mM L-NAME and 1 µM Ang II, remove media, wash with 1X DPBS and fix cells with 4% paraformaldehyde in 1X DPBS for 10 min at room temperature, and rinse cells with 1X DPBS three times.
  2) Incubate with blocking buffer for 30 min at room temperature.
  3) Rinse cells with 1X DPBS three times.
  4) Incubate cells with 1X rhodamine phalloidin (200 times diluted from stock) in blocking buffer for 30 min at room temperature.
  5) Rinse cells with 1X DPBS three times.
  6) Stain the nuclei DAPI solution for 5 min at room temperature.
  7) Rinse slides with 1X DPBS and mount coverslips face down on a slide using slide mounting media.
  8) Observe with a fluorescent microscope with a TRITC filter cube to detect F-Actin and a DAPI filter cube to detect nuclei.
d) Endothelial (CD31) and Mesenchymal (Vimentin) marker staining—(time for experiment—2 days)
  1) After 4 days of EndoMT induction by either 10 ng/ml of TGF-β1 or 1 mM L-NAME and 1 µM Ang II, remove media, wash with 1X DPBS and fix cells with 4% paraformaldehyde in 1X DPBS for 10 min at room temperature, and rinse cells with 1X DPBS three times.
  2) Incubate with blocking buffer for 30 min at room temperature.
  3) Rinse cells with 1X DPBS three times.
  4) Incubate cells with primary antibodies in blocking buffer overnight at 4^°^C in the dark according to the manufacturer’s recommended concentration. Use CD31 and TRITC conjugated Vimentin at 1:200 dilution.
  5) Rinse cells with 1X DPBS three times.
  6) Incubate cells with a green dye conjugated secondary antibody for CD31 in blocking buffer for 1 h at room temperature in the dark according to the manufacturer’s recommended concentration. Use donkey anti-goat IgG at 1:200 dilution.
  7) Rinse cells with 1X DPBS three times.
  8) Stain the nuclei DAPI solution for 5 min at room temperature.
  9) Rinse slides with 1X DPBS and mount coverslips face down on a slide using slide mounting media.
  10) Observe with a fluorescent microscope with a FITC filter cube to detect CD31, a TRITC filter cube to detect Vimentin, and a DAPI filter cube to detect nuclei.
  11) For evaluating the endothelial and mesenchymal marker expression of the recovery HUVEC cells, Plate 1.0 × 10^4^ to 3.0 × 10^4^ HUVEC cells onto each well of an 8-chambered slide and follow steps 1 to 4. Then proceed with step 5d.

#### 2.1.7 Cell Viability Assay During Endothelial-to-Mesenchymal Transition and Recovery

a) Plate 4.0 × 10^4^ to 6.0 × 10^4^ HUVEC cells onto each well of a 24-well plate, such that the cells will be ∼80% confluent in 24 h.
b) On days 1, 4, and 10 of the EndoMT and recovery assay, perform the cell viability assay as follows
  1) Aspirate the existing media from the culture plates wells carefully.
  2) Dispense 1 ml of alamarBlue working solution to each well of a 24-well plate.
  3) Incubate cells for 2 h at 37^°^C in a CO_2_ incubator.
  4) Use empty wells without cells containing 1 ml of alamarBlue working solution as negative or blank.
  5) After 2 h, transfer 100 µl of the media to a clearly marked 96-well plate and measure the fluorescence at an excitation and emission wavelength of 560 and 590 nm, respectively, using a microplate reader.
  6) Presence of cells can be viewed by the metabolic reduction of the alamarBlue-containing media, which changes from a dark blue to purple-pink.
  7) Plot the relative fluorescence of the cells at different time points to get a cell viability plot.

#### 2.1.8 Endothelial Functionality Assays of Human Umbilical Vascular Endothelial Cells After Recovery From Endothelial-to-Mesenchymal Transition

a) Nitric oxide (NO) production—(time for experiment—1 day).
  1) After 4 days of EndoMT induction following steps 2 to 4 and recovery from EndoMT following step 5, in an 8-chambered slide, wash the HUVECs with HBSS with calcium and magnesium three times.
  2) Incubate cells with 200 µl of 8 µM of DAF-FM™ diacetate solution for 30 min at 37^°^C in a CO_2_ incubator.
  3) Wash the cells with HBSS with calcium and magnesium three times.
  4) Replace with fresh EGM^TM^-2 media.
  5) Incubate cells for 15 min at 37°C in a CO_2_ incubator.
  6) Observe with a fluorescent microscope with a FITC filter cube to detect NO.
b) Acetylated Dil LDL uptake—(Time for experiment—1 day).
  1) After 4 days of EndoMT induction following steps 2 to 4 and recovery from EndoMT following step 5, in an 8-chambered slide, wash the HUVECs with HBSS with calcium and magnesium three times.
  2) Incubate cells with 200 µl of DiI AcLDL working solution for 4 h at 37^°^C in a CO_2_ incubator.
  3) Wash the cells with HBSS with calcium and magnesium three times.
  4) Observe with a fluorescent microscope with a TRITC filter cube to detect DiI AcLDL.
c) Angiogenesis assay—(Time for experiment—1 day)
  1) Prepare HUVECs following steps 2, 3, 4, and 5.
  2) Thaw Matrigel by removing the Matrigel from −20^°^C or −80^°^C freezer and place it in a refrigerator at 4^°^C.
  3) Label wells of 48-well plate as follows: Control HUVEC, HUVEC + TGF-β1, HUVEC + L-NAME/Ang II, HUVEC + TGF-β1+ Recovery and HUVEC + L-NAME/Ang II + Recovery.
  4) Place a vial of completely thawed Matrigel and labeled a 48-well plate on ice in a laminar flow hood. Mix the vial thoroughly by inverting a few times. Load 140 µl of Matrigel per well of a 48-well plate while avoiding air bubbles.
  5) Transfer the 48-well plate to a cell culture incubator and incubate it at 37^°^C for 30 min to allow the Matrigel to gel.
  6) Harvest single-cell suspension using standard methods as mentioned in steps 2 b to f.
  7) Resuspend cell pellet in appropriate medium (Control—EGM^TM^-2, HUVEC + TGF-β1/L-NAME + Ang II—EBM^TM^-2, HUVEC + TGF-β1/L-NAME/Ang II + Recovery—EGM^TM^-2) at a concentration of 7.5 × 10^5^ cells per ml.
  8) Gently dispense 400 µl (30,000 cells) per well of the single-cell suspensions to corresponding labeled wells of a 48-well plate on top of the gelled Matrigel.
  9) Incubate the 48-well plate at 37^°^C in a CO_2_ incubator for a period of 4–8 h or until the desired result is achieved. Examine the plate every hour for tube formation under an inverted microscope with ×4 or ×10 objective.
  10) Once tube formation is observed, photograph the tubular network in the wells using a digital camera attached to the inverted microscope with ×4 or ×10 objective.

#### 2.1.9 Image Analysis—(Time for Experiment—Variable)

a) CD31 and Vimentin Fluorescent intensity (FI) quantification.
  1) Open Image J (Schneider et al., 2012) software.
  2) Click “File,” and then click “Open,” to open the file you want to quantitate. Or drag and drop the file to the ImageJ window to open.
  3) Click “Plugins,” and choose “Analyze and then click “RGB Measure”.
  4) Save the “Results” window in a desired file location.
  5) Depending on the fluorescent channel being measured (red, green, or blue), use the integrated density values for FI and plot FI for different groups.
b) NO production and Acetylated LDL uptake FI quantification.
  1) Open ImageJ software and open the appropriate file using step 8a 2.
  2) Click on “Analyze” tab and choose the “Set measurements…” option. Check the option for “Integrated Density” and click ok.
  3) Click the “Freehand selections” button located below the “Image” tab and use the drawing tool to mark cells and choose the region of interest (ROI) to be quantitated.
  4) After marking the desired area, click on “Analyze” tab and choose the “Measure” option. Repeat for multiple measurements.
  5) Save the “Results” window in a desired file location.
  6) Depending on the fluorescent channel being measured (red, green, or blue), use the integrated density values for FI and plot FI for different groups.

#### 2.1.10 Statistics

a) The results are presented as means ± standard deviations (SD) of *n* = 3, unless specified otherwise. Statistics was calculated using GraphPad Prism 8 (refer to GraphPad tutorials for detailed instructions on analysis). Difference between means were analyzed using the one-way analysis of variance (ANOVA) and posthoc Tukey test to compare group means. *p* < 0.05 is considered as significant difference.

## 3 Representative Results

### 3.1 Nω-Nitro-L-Arginine Methyl Ester Hydrochloride/Angiotensin II Induces Endothelial-to-Mesenchymal Transition Comparable to Transforming Growth Factor-β1

**FIGURE 2:**
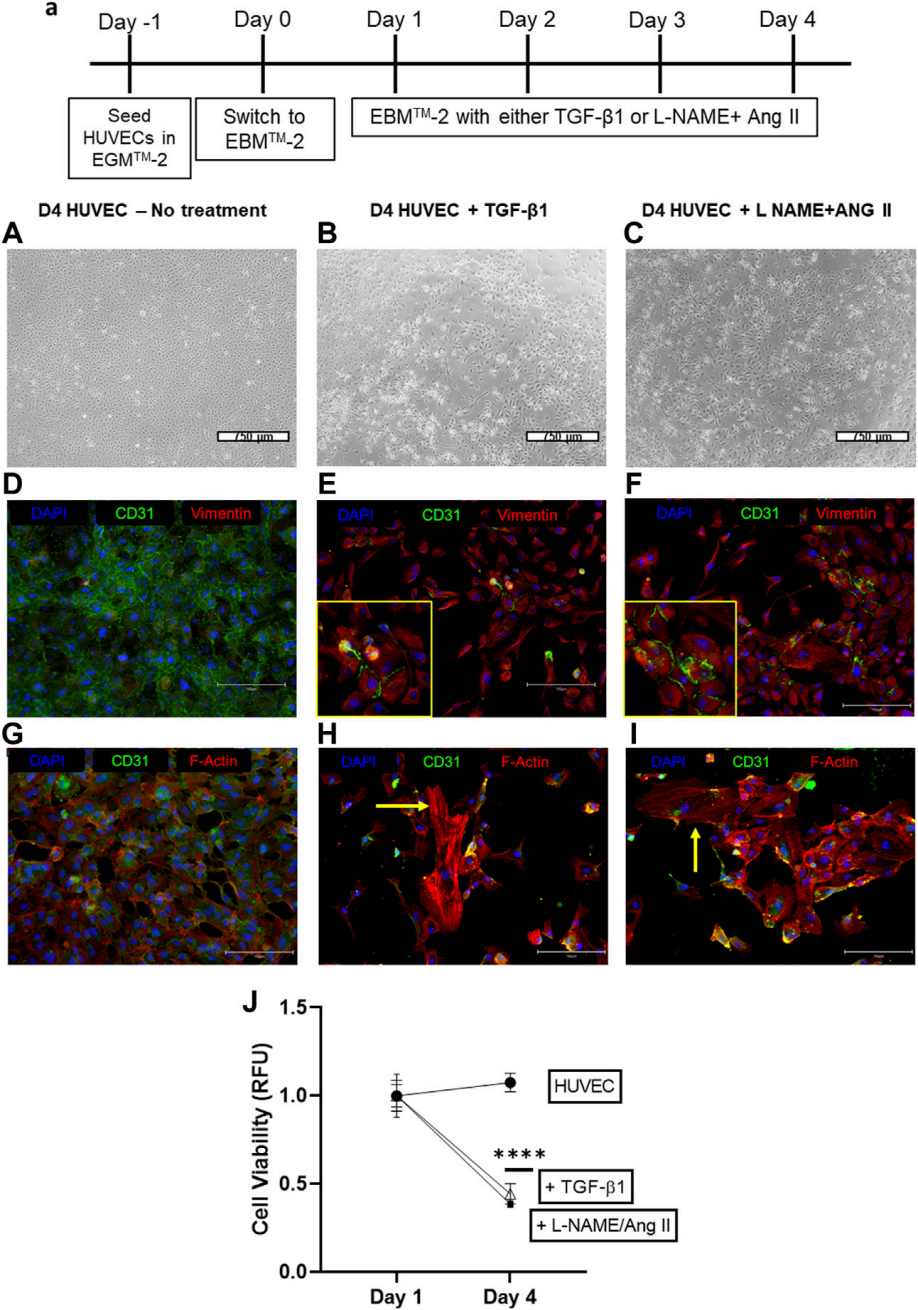
Induction of EndoMT in HUVECs cells by TGF-β 1 or L-NAME + Ang II. **(A)** Representative scheme for EndoMT **(A–C)** Representative images of morphological changes of HUVECs after TGF-β or L-NAME + Ang II treatment; Scale bar: 750 μm; **(D–F)** Representative images exhibiting the presence of CD31 and Vimentin positive cells indicating the EndoMT process; Yellow insets represent the zoomed-in regions with CD31^+^ and Vimentin + cells that indicate EndoMT; Scale bar: 150 µm **(G–I)** Representative images demonstrating F-actin reorganization of HUVECs during TGF-β/L-NAME + Ang II treatment; Yellow arrows indicate actin reorganization in the form of stress fibers; Scale bar: 150 µm **(J)** Cell viability was measured using the alamarBlue assay at different time points during EndoMT.

HUVEC cells which are treated with a combination of L-NAME + Ang II lose their characteristic cobblestone-like morphology and begin to form spindle-shaped mesenchymal-like cells (Figure 2C) compared to control cells (Figure 2A). This cell transition is similar to EndoMT induction seen when HUVEC cells are treated with TGF-β1 (10 ng/ml) (Figure 2B).

After treatment with either TGF-β1 or L-NAME + Ang II for 4 days, immunofluorescence analyses demonstrate decreased expression of CD31 and increased expression of Vimentin (Figures 2E,F) when compared to control HUVEC (Figure 2D). Presence of dual-stained cells (CD31^+^ and Vimentin+), a marker of EndoMT was also observed at this time point (Figures 2E,F insets). Treatment with EndoMT inducing agents also resulted in the reorganization of cytoskeleton and formation of stress fibers as exhibited by F-actin staining (Figures 2H,I), when compared to the control group (Figure 2G). These effects suggest the acquisition of a mesenchymal-like state through the EndoMT process (Medici and Kalluri, 2012). Furthermore, the presence of the stressor while EndoMT is induced also affected the viability of the cells as demonstrated using the alamarBlue assay. A significant decrease in cell viability was observed between days 1 and 4 for cells cultured with TGF-β1 or L-NAME + Ang II compared to control HUVECs (Figure 2J).

### 3.2 Endothelial-to-Mesenchymal Transition Induced by Transforming Growth Factor-β1/Nω-Nitro-L-Arginine Methyl Ester Hydrochloride/Angiotensin II Is Reversible

**FIGURE 3:**
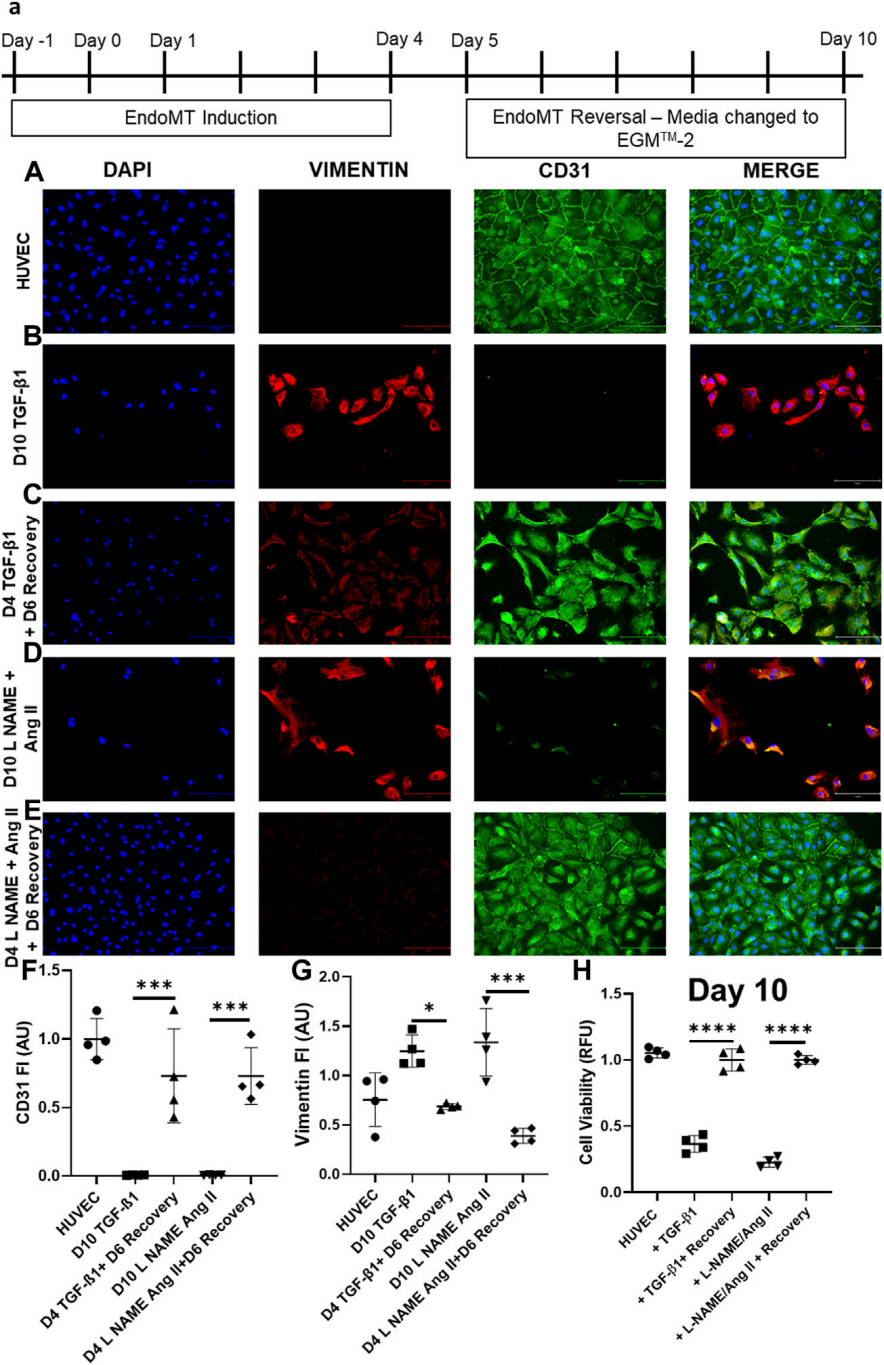
EndoMT is reversible in HUVECs when TGF-β1 or L-NAME + Ang II stress is removed. **(A)** Representative scheme for EndoMT and its reversal. Representative images of immunofluorescent stained cells for CD31, Vimentin, and DAPI in **(A)** HUVEC-No treatment, **(B)** HUVEC treated with TGF-β1 for 4 days **(C)** HUVECs treated with TGF-β1 for 4 days followed by 6 days culture in complete EGMTM-2 medium **(D)** HUVEC treated with L-NAME + Ang II for 4 days **(E)** HUVECs treated with L-NAME + Ang II for 4 days followed by 6 days culture in complete EGMTM-2 medium; Scale bar: 150 µm **(F,G)** Fluorescent intensity of CD31 and Vimentin stained cells in panel **(A–E)** calculated with Image J (*n* = 4); Data presented as ×20 microscopic field measurements. **(H)** Cell viability was measured using the AlamarBlue assay on day 10 of EndoMT and day 6 of recovery (*n* = 4).

In HUVEC cells, the decreased CD31 and increased vimentin are reversed, when the EndoMT inducing agents (TGF-β1 or L-NAME + Ang II) are removed after day 4. Following EndoMT, when TGF- 1 or L-NAME + Ang II containing media was replaced with complete EGM^TM^-2 media, the cells exhibit an increase in CD31 expression and a decrease in Vimentin expression at day 6 following the removal of induction agents (Figures 3C,E,F). Whereas in the groups which were maintained with TGF-β1 or L-NAME + Ang II, cells continued to maintain a mesenchymal state (decreased CD31 and increased Vimentin expression compared to control HUVECs) as shown in Figures 3B,D also confirmed semi-quantitatively using florescent intensity in Figures 3F,G. This reversal in marker expression is also accompanied with better cell viability compared to the cells that had the EndoMT inducing agents maintained until day 10. (Figure 3H).

### 3.3 Human Umbilical Vascular Endothelial Cells Exhibit Functional Recovery After Endothelial-to-Mesenchymal Transition Reversal

**FIGURE 4:**
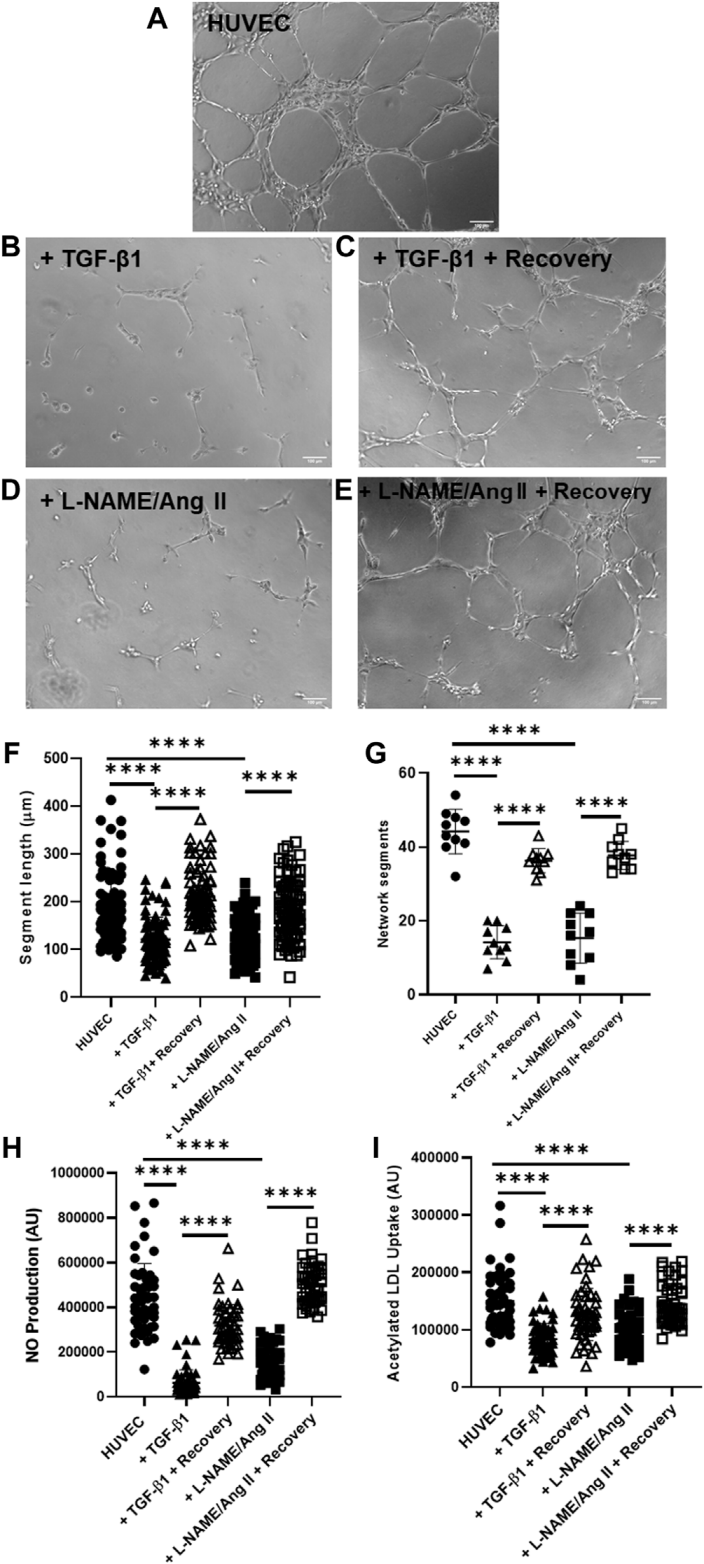
EndoMT reversal after TGF-β1 or L-NAME + Ang II stress removal restores endothelial function in HUVECs. Representative images of Matrigel® network segment assays for control HUVECs **(A)**, HUVECs after day 4 of EndoMT (with TGF-β1 or L-NAME + Ang II) **(B,D)**, and recovered HUVECs (day 6 after removal of EndoMT inducing agents) **(C,E)**; Scale bar—100 µm **(F)** shows quantification of segment length for *n* = 85 segments **(G)** shows quantification of network segments for *n* = 10 fields of view. Endothelial function was also assessed with fluorescence assays for **(H)** NO production using DAF-FM (*n* = 50 individual cells) and **(I)** acetylated LDL uptake (*n* = 50 individual cells), with single-cell fluorescence quantified.

Endothelial cell–specific functionality was evaluated using three methods. Removal of TGF-β1 or L-NAME + Ang II improved network formation in Matrigel^®^ tube formation assay (Figures 4C,E) when compared to HUVECs treated with TGF-β1 or L-NAME + Ang II for 4 days (Figures 4B,D). Recovered HUVECs exhibited significantly increased segment length (Figure 4F) and network segments (Figure 4G). Improved NO generation (Figure 4H) and acetylated LDL uptake (Figure 4I) was also observed in the recovered HUVECs compared to the cells with the induction agents (day 4 EndoMT group). These data show that the functional characteristics of cells were impaired when stimulated by the induction agents and the functionality of the endothelial cells was restored once reversed.

## 4 Discussion

TGF-β1 has been established as a promoter of EMT in the context of cancer metastasis and many *in vitro* platforms exist to study EMT (Zeisberg et al., 2007). L-NAME and Ang II have been known to induce EndoMT independently (at certain concentrations that served as reference for our preliminary testing) (Mihira et al., 2012; Gao et al., 2020; Tombor et al., 2021) but have not gained widespread attention for laboratory-based assays as a platform to study the impact of pharmacotherapies. With recent attention driven towards the role of EndoMT in various cardiovascular diseases including heart failure, we addressed such a need by creating an *in vitro* model of EndoMT induction and reversal using agents that have been validated to cause heart failure in animal models (Wang et al., 2020). While both agents can induce transitioning independently, we chose the combination of the agents to recapitulate our mouse model where fibrosis is more extensive than other mouse models of heart failure. The results presented here demonstrate that the combination of L-NAME + Ang II induced EndoMT in HUVEC cells. While many established therapies have shown observational data in the reduction in fibrosis in animal and human studies, none have been specifically associated with impacting the EndoMT pathway. On the contrary, studies in renal cells have shown that the EMT pathway could be mediated by receptors not targeted by current therapies (Grande et al., 2015). By establishing an *in vitro* model, we provide a platform that can be used to target a pathway which has been shown to mediate fibrosis in various pathological states including heart failure (Zeisberg et al., 2007; Wang et al., 2020; Tombor et al., 2021).

HUVEC viability was measured with alamarBlue (Sarig et al., 2018; Krishnamoorthi et al., 2020) and the viability trend we observed in our work is comparable to the endothelial viability studied with the presence of TGF-β1, L-NAME, and Ang II as shown by others (Hirai and Kaji, 1992; Mocellin et al., 2004; Du et al., 2016; Zhang et al., 2020). While reversibility of markers of EndoMT in endothelial cells has been reported previously, this reversibility has not been previously characterized as a functional reversal. Our results exhibit that EndoMT induced with L-NAME + Ang II is reversible, and HUVECs not only display change in endothelial and mesenchymal marker expression pattern upon EndoMT reversal but also regain endothelial-specific functionality. This endothelial functionality assessment of cells undergoing both EndoMT and its reversal could be used to study and identify interventional molecules to target different stages of EndoMT and the resulting pathology. This observation of EndoMT could have similarity to the *in vivo* “natural recovery” we observe in our HF mouse model as well, where removal of HF inducing agents results in reversal of heart failure phenotype (Wang et al., 2020). While TGF-β1 is an established agent to induce EMT *in vitro*, our platform described above can have the advantage to be congruous with *in vitro* experiments where L-NAME and angiotensin II combination has been shown to induce heart failure. We used HUVEC cells as representative of the various endothelial cells but acknowledge that endothelial cells of various organ vascular beds could have different properties. The method presented here may be subject to limitations in terms of the specifics of inducing agent concentration, culture period, and medium composition, as they might need to be tailored to study endothelial cells of different origins, as described previously (Paranya et al., 2001; Zeisberg et al., 2007; Hashimoto et al., 2010; Mihira et al., 2012; Krizbai et al., 2015; Tombor et al., 2021), for different applications. Also, an impact on cell viability due to the stress of transitioning can confound the ability to know the direct toxicity of any molecule studied. We suggest that a control experiment with the agent could mitigate this confounder.

In conclusion, we present a protocol to induce EndoMT with L-NAME + Ang II and its reversal *in vitro*. This method can be used to study the impact of potential therapeutic interventions (pharmaceutical molecules, genetic manipulations using viral and nano vectors, etc.) on the EndoMT mechanism. We expect that this protocol will serve as an avenue for investigating EndoMT and lead to establishing approaches to either inhibit EndoMT or promote MEndoT.

## Data Availability

The original contributions presented in the study are included in the article/Supplementary Material, further inquiries can be directed to the corresponding author.
